# Continued smoking in African American cancer survivors: The Detroit Research on Cancer Survivors Cohort

**DOI:** 10.1002/cam4.3368

**Published:** 2020-08-21

**Authors:** Carly M. Malburg, Juliana Fucinari, Julie J. Ruterbusch, David M. Ledgerwood, Jennifer L. Beebe‐Dimmer, Ann G. Schwartz, Michele L. Cote

**Affiliations:** ^1^ Department of Oncology Wayne State University School of Medicine Detroit MI USA; ^2^ Department of Psychiatry and Behavioral Neurosciences Substance Abuse Research Division Wayne State University School of Medicine Detroit MI USA; ^3^ Population Studies and Disparities Research Program Detroit MI USA

**Keywords:** breast cancer, cancer survivorship, colon cancer, health disparities, lung cancer, prostate cancer, smoking cessation

## Abstract

Tobacco cessation among those recently diagnosed with cancer is important to improve their prognosis, yet, many cancer survivors continue to smoke. The epidemiology of tobacco use differs by race and ethnicity, and limited cessation research has been conducted in African American (AA) populations. Here, we assess demographic and clinical variables associated with continued smoking in AAs after a cancer diagnosis. The Detroit Research on Cancer Survivors study is a cohort comprised of AA cancer survivors with breast, prostate, lung, and colorectal cancers. Detroit Research on Cancer Survivors data were utilized from survivors who completed their baseline survey within 18 months of cancer diagnosis (n = 1145); 18% (n = 356) reported smoking at the time of cancer diagnosis, and 57% of these (n = 203) continued to smoke after their diagnosis. Logistic regression models were used to assess factors associated with continued smoking. Living with a smoker (odds ratio [OR] = 2.78, 95% confidence interval [CI]: 1.64, 4.70), higher cumulative years of smoking (OR = 1.03, 95% CI: 1.01, 1.05, for each year), and a prostate cancer diagnosis (OR = 7.35, 95% CI: 3.89, 13.89) were all associated with increased odds of continued smoking. Survivors with higher social well‐being scores (measured by the Functional Assessment of Cancer Therapy, a quality of life assessment) were more likely to quit smoking after diagnosis (OR = 0.96, 95% CI: 0.93, 1.00). These findings highlight the continued need for personalized cessation strategies to be incorporated into treatment plans for cancer survivors.

## Graphical abstract

African Americans (AA) cancer survivors who smoked at diagnosis were less likely to report quitting if they lived with another smoker, had higher cumulative years of smoking, and a prostate cancer diagnosis. Individuals with higher social well‐being scores were more likely to quit smoking.

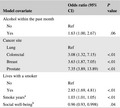

**Key points**

- Data from the Detroit Research on Cancer Survivors cohort study were used to examine factors related to smoking continuation after a cancer diagnosis in African American cancer survivors.
- We found that survivors who were living with a current smoker, had a long personal smoking history, and had a nonlung cancer diagnosis more likely to continue to smoke.
- Overall, our research highlights that smoking cessation interventions should consider these factors when counseling cancer survivors.

## 1 INTRODUCTION

Tobacco smoking has been declining over the last several decades in the United States, yet 60% of people diagnosed with cancer report being current smokers, having quit within a year of diagnosis, or are former smokers.^1^, ^2^, ^3^ Among current smokers with cancer, it is estimated that only 50% stop smoking after diagnosis despite the benefit tobacco cessation brings to an individual's health.^1^, ^2^ Cancer survivors are more likely to continue to smoke if they have prolonged years of smoking, live with or have a caregiver who smokes, and if they do not perceive smoking cessation as necessary to improve their health and quality of life (QOL).^1^, ^2^, ^4^, ^5^ Other factors that may affect smoking cessation in cancer survivors include being from an underrepresented population, alcohol use, obesity, and socioeconomic status.^1^, ^2^, ^4^, ^5^, ^6^ Generally, those who are most likely to continue smoking after diagnosis have lower incomes and are less educated compared to those who report successful smoking cessation.

Continued smoking puts survivors at risk of reporting an overall poorer QOL when compared to cancer survivors who have never smoked or have successfully quit smoking.^3^, ^4^, ^6^, ^7^, ^8^, ^9^, ^10^, ^11^ Additionally, cancer patients who are current smokers at the time of diagnosis and successfully quit smoking see an improvement in the all‐cause mortality rate compared to patients who continued to smoke.^12^ Cancer survivors who continue to smoke have greater adverse reactions to treatment, are at a greater risk of developing a secondary smoking‐related malignancy, and are at a greater risk of disease progression or relapse.^3^, ^6^, ^7^, ^8^, ^9^, ^10^, ^13^ Warren et al. found that the overall mortality rate among all cancer patients who continued smoking was 20% higher than patients who had recently quit (defined as those who stopped smoking within 1 year prior to diagnosis).^14^ Schootman et al. reported that African American (AA) cancer survivors had an overall higher prevalence of poor health status, lower QOL, and greater likelihood of being a current smoker compared to cancer survivors from other racial/ethnic groups.^15^ In 2015, The National Comprehensive Cancer Network published guidelines for smoking cessation in cancer survivors as part of supportive care, but it is clear many survivors continue to smoke.^16^

In the United States, smoking, smoking‐related disease, and multiple quit attempts are more prevalent among individuals from underrepresented minority populations as well as those from lower socioeconomic status groups, compared to non‐Hispanic white (NHW) populations or those in higher socioeconomic groups.^1^, ^2^ Specifically, populations that are more likely to have higher rates of smoking are those with no formal education beyond high school, racial minorities, veterans, and non‐married individuals.^11^, ^17^, ^18^ The 2008 National Health Interview Survey reported that AAs are more likely to take up smoking later in life and smoke fewer cigarettes, but are more likely to experience higher tobacco dependencies.^11^ In addition, AAs attempt to quit smoking more often, are less successful in their quit attempts, and are at a greater risk of developing smoking‐related diseases when compared to NHW populations.^9^, ^11^, ^15^ Further, AA are more likely to use menthol cigarettes, which are thought to be more addictive than nonflavored cigarettes.^9^, ^11^ Use of menthol cigarettes could predispose AAs to become more dependent on nicotine, ultimately leading to the development of smoking‐related diseases.^10^, ^14^, ^17^

Racial and ethnic minority populations have been underrepresented in cancer survivorship research. Given that AAs tend to have the poorest prognosis for most cancers, we examine factors associated with continued smoking after a cancer diagnosis in a cohort of AA cancer survivors.

## 2 METHODS AND MATERIALS

The Detroit Research on Cancer Survivors (ROCS) study, a large population‐based cohort of AA cancer survivors, was used for this analysis. This survivorship cohort includes individuals who were diagnosed with lung, breast, prostate, or colorectal cancer. Potential participants were identified through the Metropolitan Detroit Cancer Surveillance System (MDCSS), a population‐based cancer registry that covers all residents of three counties in Southeastern Michigan (Wayne, Oakland and Macomb), and a founding member of the Surveillance, Epidemiology, and End Results (SEER) program.

The Detroit ROCS study includes patients with first primary cancers (diagnosed from January 1, 2013 and continuing through December 31, 2021) whose self‐reported race was Black or AA. Detailed methods have been published elsewhere^19^ but briefly, cases are identified from MDCSS and after providing informed consent, answer a structured survey. Questions include medical history, family history of cancer, lifestyle factors, access to health care, healthy literacy, health‐related QOL (presence of depression or anxiety), and social support. Participants are also queried regarding past and current cigarette smoking status, including smoking status at the time of the cancer diagnosis, duration of smoking (in years), age started smoking, smoking cessation, and whether other members in the household smoke cigarettes.

Demographic variables in this analysis included: age at diagnosis, age at baseline ROCS survey, sex, income (<$40 000 vs ≥$40 000), education (high school or less vs some college or more), and marital status (married/living with partner, never married, or widowed/divorced/separated). Health history variables included body mass index based on reported weight 1 year prior to cancer diagnosis and at survey, categorized as ≥30 kg/m^2^ (obese) or <30 kg/m^2^ (not obese). Also included were the number of reported comorbid conditions (coded ordinally from none to four or more), SEER Summary Stage (local, regional, and distant), and self‐reported treatment (surgery, chemotherapy, and radiation therapy). Health‐related quality of life (HRQOL) measures in the ROCS survey include both the Functional Assessment of Cancer Therapy (FACT) questionnaire and Patient‐Reported Outcomes Measurement Information System (PROMIS) questionnaire for anxiety and depression (short form 4a). The FACT is a specific assessment of QOL for cancer patients and is composed of 4 sub‐scores: functional well‐being, emotional well‐being, physical well‐being, and social well‐being, with higher scores indicating better QOL. The FACT general assessment was first validated in 1993,^20^ and has since been translated into various languages, validated in diverse populations, and specific versions for different cancer sites have been developed.^21^, ^22^, ^23^

Patient‐Reported Outcomes Measurement Information System is a U.S. National Institutes of Health Common Fund initiative which has developed a bank of questionnaires available to researchers to measure function that have been validated in diverse populations.^24^ Patient‐Reported Outcomes Measurement Information System responses are normalized to the general U.S. population with an average score of 50, with higher PROMIS scores indicating higher levels of anxiety/depression.

The Detroit ROCS study is ongoing and will enroll over 5500 AA cancer survivors. Data from the first 2500 ROCS participants enrolled in the study were used in this analysis. Participants who completed the ROCS baseline survey later than 18 months after receiving a cancer diagnosis were excluded from data analysis (n = 1355), as well as those missing smoking data (n = 6). The decision to exclude these patients was made so that patient survey responses used in this analysis would, as accurately as possible, reflect smoking behaviors influenced by the patient’s cancer diagnosis and treatment. The final analytic data set included 1162 cancer survivors diagnosed from 2013 to 2019.

### 2.1 Data analysis

All data analyses were performed using SAS Software v9.4 for Windows (Copyright ©2002‐2012 SAS Institute Inc.). The distribution of demographic, clinical, smoking habit, and HRQOL variables were summarized by smoking status at time of cancer diagnosis and at baseline ROCS survey. Differences in the distribution of these variables by smoking status at baseline ROCS survey were assessed for those who reported smoking at the time of diagnosis. Frequency differences and 95% confidence intervals were also calculated to indicate difference in percentage of individuals who continued to smoke after diagnosis compared to those that quit postdiagnosis.

Odds ratios (OR) and 95% confidence intervals (95% CI) were estimated using logistic regression models to examine potential associations between demographic, clinical, and HRQOL factors and reported smoking at baseline ROCS survey compared to those who reported quitting between their cancer diagnosis and survey. Variables of interest were included a priori based on review of the current literature. Multivariate models were constructed using a modified stepwise approach. All variables were initially included, then, variables were excluded until only variables that were statistically significant at α=0.05 remained. Variables were, in turn, reintroduced into the model for identifying any variables that, once reintroduced, caused any β estimates to change by 10% or more.

## 3 RESULTS

Of the 1,162 survivors included in the analysis, 497 (43%) reported never smoking, 467 (40%) were former smokers, and 202 (17%) were current smokers at baseline ROCS survey. Table 1 includes survivors who reported smoking at diagnosis, stratified by those who quit within 18 months of diagnosis, and those who continued to smoke. The median age at diagnosis for current smokers and those who quit after diagnosis was 59.0. More men continued to smoke after diagnosis compared to women (*P* < .01). Education, income level, and marital status did not differ between those who quit after diagnosis and those who continued to smoke (*P* = .83, .44, and .73, respectively). Obesity before diagnosis or at the time of interview did not affect smoking status (*P* = .77 and .50, respectively), nor did the number of comorbid conditions (*P* = .48). Use of alcohol in the last month, however, was more common among those who continued to smoke postdiagnosis (*P* < .01). Number of years of smoking and age at smoking initiation were not associated with smoking continuation (*P* = .20 and .48, respectively), but living with a smoker in the household was associated with continuing to smoke postdiagnosis (*P* < .01)

**TABLE 1:** Select clinical and demographic variables of ever smokers by smoking status at diagnosis and baseline ROCS survey among African American cancer survivors of Metropolitan Detroit (diagnosed 2013‐2019)

Characteristic	Smoking Status after diagnosis		
	Quit after cancer diagnosis	Current smoker	*P*‐value^a^
	(N = 158)	(N = 202)	
Demographics			
Age (at survey)			
Mean (SD)	59.8 (8.6)	60.0 (7.7)	.75
Median (range)	59.5 (44.0)	60.0 (45.0)	
Age (at diagnosis)			
Mean (SD)	58.9 (8.6)	59.0 (7.6)	.78
Median (range)	59.0 (44.0)	59.0 (45.0)	
Gender			
Male	70 (44.3)	118 (58.4)	<.01
Female	88 (55.7)	84 (41.6)	
Education			
High school diploma/GED or less	96 (60.8)	125 (61.9)	.83
Some college or more	62 (39.2)	77 (38.1)	
Income			
<$40 000	140 (88.6)	184 (91.1)	.44
$40 000+	18 (11.4)	18 (8.9)	
Marital status			
Married/living with partner	43 (31.6)	54 (32.9)	.73
Never married	43 (31.6)	45 (27.4)	
Widowed, divorced, separated	50 (36.8)	65 (39.6)	
Health			
Pre‐dx obesity			
Not obese	104 (65.8)	130 (64.4)	.77
Obese	54 (34.2)	72 (35.6)	
Current obesity			
Not obese	109 (69.0)	146 (72.3)	.50
Obese	49 (31.0)	56 (27.7)	
Comorbidity count			
None	15 (9.6)	13 (6.4)	.48
1	23 (14.7)	43 (21.3)	
2	25 (16.0)	40 (19.8)	
3	32 (20.5)	33 (16.3)	
4 or more	61 (39.1)	73 (36.1)	
Alcohol use			
Consumed alcohol in past month	70 (44.3)	116 (57.4)	.01
Did not consume alcohol	88 (55.7)	86 (42.6)	
Smoking habits			
Years smoked			
Mean (SD)	37.2 (12.3)	39.1 (11.0)	.20
Median (range)	40.0 (55.0)	40.0 (60.0)	
Age at beginning to smoke			
Mean (SD)	17.9 (5.4)	17.8 (6.1)	.48
Median (range)	17.0 (38.0)	16.5 (55.0)	
Smoker in household			
Yes	40 (25.5)	86 (43.0)	<.01
No	117 (74.5)	114 (57.0)	
Cancer site			
Breast	42 (26.6)	51 (25.3)	<.01
Prostate	30 (19.0)	91 (45.1)	
Lung	69 (43.7)	37 (18.3)	
Colorectal	17 (10.8)	23 (11.4)	
Stage at diagnosis			
Local	62 (39.2)	109 (54.2)	.01
Regional	59 (37.3)	56 (27.9)	
Distant	37 (23.4)	36 (17.9)	
Treatment			
Surgery			
Yes	96 (60.8)	108 (55.4)	.31
No	62 (39.2)	87 (44.6)	
Chemotherapy			
Yes	89 (56.3)	71 (36.2)	<.01
No	69 (43.7)	125 (63.8)	
Radiation			
Yes	77 (49.4)	98 (50.3)	.88
No	79 (50.6)	97 (49.7)	
Quality of life measures			
Total FACT score			
Mean (SD)	71.6 (18.2)	70.6 (18.8)	.77
Median (range)	72.0 (85.0)	73.0 (92.0)	
FACT sub‐scores			
Physical well‐being			
Mean (SD)	19.1 (6.6)	19.2 (6.6)	.99
Median (range)	72.0 (85.0)	19.0 (27.0)	
Social well‐being			
Mean (SD)	19.9 (6.2)	18.2 (6.6)	.02
Median (range)	21.0 (28.0)	19.0 (23.0)	
Emotional well‐being			
Mean (SD)	18.1 (4.8)	18.1 (5.0)	.84
Median (range)	19.0 (20.0)	19.0 (23.0)	
Functional well‐being			
Mean (SD)	14.3 (6.9)	15.0 (6.9)	.30
Median (range)	14.0 (28.0)	14.0 (28.0)	
PROMIS score: anxiety			
Mean (SD)	52.7 (10.5)	52.5 (11.0)	.74
Median (range)	53.7 (41.3)	52.5 (41.3)	
PROMIS score: depression			
Mean (SD)	50.7 (9.5)	50.4 (9.9)	.65
Median (range)	51.8 (32.3)	51.8 (34.7)	

Abbreviations: GED, General Educational Development test; FACT, Functional Assessment of Cancer Therapy; PROMIS, Patient‐Reported Outcomes Measurement Information System.

^a^ *P*‐values for comparisons between those who quit after diagnosis and current smokers were calculated from chi square tests for categorical variables, Cochran Armitage test for ordinal variables, and Wilcoxon Log Rank tests for continuous variables.

Table 1 also describes clinical factors and psychosocial measures among smokers that did and did not quit smoking after a cancer diagnosis. Individuals with prostate cancer and those with earlier stage cancers at diagnosis were both associated with continued smoking (both *P* < .01), while those who received chemotherapy were more likely to quit after their diagnosis (*P* < .01). Self‐reported quality of life measures did not differ between those who continued to smoke after diagnosis and those who quit, with the exception of the FACT social well‐being sub‐score, which was lower among those who continued to smoke (*P* = .02).

Table 2 shows factors associated with continued smoking, each adjusted for alcohol use in the last month, cancer site, living with a smoker, number of years of smoking, and reported social well‐being. Among those who reported smoking at diagnosis, a one‐point increase in reported social well‐being coincided with 4% lower odds of continued smoking (OR = 0.96, 95% CI: 0.90, 1.00) after adjustment. Those who reported living with a smoker had nearly three times the odds of continued smoking than those not living with a smoker (OR = 2.85, 95% CI: 1.69, 4.81). Each additional year of smoking prior to diagnosis was associated with an average of 3% higher odds of continued smoking (OR = 1.03, 95% CI: 1.01, 1.05). Compared to lung cancer survivors, all other cancer sites had higher odds of continued smoking, with the highest among prostate cancer patients, with over sevenfold the odds of continued smoking (OR = 7.35, 95% CI: 3.89, 13.89; Table 2).

**TABLE 2:** Association of clinical and demographic variables with continued smoking after a cancer diagnosis among African American cancer survivors in Metropolitan Detroit (diagnosed 2013‐2019)

Model covariate	Odds ratio (95% CI)	*P* value
Alcohol within the past month		
No	Ref	
Yes	1.63 (1.00, 2.67)	.06
Cancer site		
Lung	Ref	
Colorectal	3.08 (1.32, 7.15)	<.01
Breast	3.63 (1.87, 7.05)	<.01
Prostate	7.35 (3.89, 13.89)	<.01
Lives with a smoker		
No	Ref	
Yes	2.85 (1.69, 4.81)	<.01
Smoke years^a^	1.03 (1.01, 1.05)	<.01
Social well‐being^b^	0.96 (0.93, 0.998)	.04

^a^ Effect size is for each additional smoke year.

^b^ Effect size is for each 1‐point increase in social well‐being FACT‐G sub‐score.

## 4 DISCUSSION

Among the Detroit ROCS cohort of AA cancer survivors, longer smoking duration and cancer diagnoses other than lung cancer were associated with continued smoking after a cancer diagnosis. These data also highlighted that living with a smoker is associated with continued smoking after a cancer diagnosis. Thus smoking cessation support should be provided not only to the cancer survivor but also to all current smokers who are in the household. It is plausible that the associations between continued smoking at diagnosis and number of smoke years, along with socioeconomic level reported by the ROCs participants (72.8% with annual income <$40 000) may be partially attributed to limited access to smoking cessation treatments (ie, Nicotine Replacement Therapy [NRT], and cessation programs). Fu et al. analyzed the use of NRT in relation to smoking cessation rates among Caucasian, AA, Asian, and Latino lifetime smokers.^25^ They reported that AA smokers were the group least likely to ever use NRT despite this group having the highest levels of nicotine dependence and were the least likely to successfully quit smoking.^25^ Simmons et al. found that external influences, such as income and additional smokers in the household, have a strong influence on a cancer patient’s smoking cessation ability.^26^ They also found that cancer patients, specifically AAs, are more likely to have fewer successful cessation attempts if they live in a smoking household.^26^

Our data analysis shows that alcohol use was also associated with continuation of smoking. While our analysis may only show a marginal association codependence between habitual smoking and alcohol use has been well‐established.^27^ Specifically, people who are dependent on tobacco are four times more likely to also be dependent on alcohol compared to the general population, and this may vary by race.^27^ Therefore, patients should be screened for cooccurring addiction, and counseling for alcohol dependence in addition to smoking cessation may be necessary.

We were able to examine smoking cessation by cancer type. Our cohort of AA cancer survivors with prostate, breast, or colorectal cancers had higher odds of continuing to smoke after diagnosis compared to those with lung cancer. Lung cancer has the lowest overall survival rate of the four sites studied, and is the most strongly associated with cigarette smoking.^8^, ^9^ Men diagnosed with prostate cancer were found to be the most likely to continue smoking, and overall have a favorable prognosis; however, smoking cessation is still critical, given cardiovascular and cerebrovascular disease are strongly associated with habitual smoking.^17^, ^28^, ^29^, ^30^ Cardiovascular disease is a leading cause of death in the United States, particularly among current smokers.^12^ AAs are more likely to die from cardiovascular disease than their white counterparts, and at younger ages.^17^, ^29^, ^30^, ^31^ Therefore, it is likely that cancer survivors, regardless of their cancer type, who continue to smoke will succumb to either cardiovascular or cerebrovascular disease if they survive their initial cancer. The period around a cancer diagnosis may represent an important window of opportunity, or a “teachable moment,” to promote tobacco cessation, for the sake of improving not only their probability of cancer survival, but also their overall health.^5^, ^32^ McBride et al. note that the impact these “teachable moments” have on successful smoking cessation can be directly related to a hospitalization or disease diagnosis.^33^ Furthermore, this rate of success is directly proportional to the severity of hospitalization or how chronic the disease diagnosis.^33^ For example, a “teachable moment” following a cancer diagnosis may have a greater impact on an individual's likelihood to quit smoking than a disease like diabetes, which is relatively common and managed at home. This rationale may partially explain why lung cancer survivors, who have poorer prognosis compared to other survivors of other cancer types, have higher quit rates.

The Detroit ROCS survey includes FACT assessment scores for each participant, and analysis showed lower odds of continuing to smoke after a cancer diagnosis for participants with higher FACT social well‐being sub‐scores. These findings are consistent with Park et al. in that the greater a cancer survivor’s quality of life and social well‐being, the more successful they will be at smoking cessation.^8^ Therefore, these findings suggest that integrating increased social support into smoking cessation programs could improve the rates of smoking cessation in this survivor population.

Our analysis had several strengths that separates our research from other smoking cessation studies. First, we utilized data from a large population‐based cohort comprised entirely of AA cancer survivors with several self‐reported quality of life and health behavior variables. This is a contemporary cohort, with all participants having received their cancer diagnosis within the last 7 years and includes several cancer sites. Second, the utilization of the MDCSS registry to identify survivors increases the likelihood that our results can be applicable to populations beyond our catchment area. This study identifies specific trends for why AA may continue to smoke after a cancer diagnosis and why it is a priority to consider smoking cessation strategies in care plans. As a whole, this research highlights a population that is frequently underrepresented in cancer survivor research.

Conversely, this research has limitations, which should be considered. Of note, smoking behavior is self‐reported by the participant, and was not biochemically validated. Additionally, details regarding smoking addiction and cessation attempts were not included in the survey. Finally, we only included individuals who completed the baseline survey within 18 months of their cancer diagnosis so results describe short‐term behaviors after a cancer diagnosis.

## 5 CONCLUSION

Among AA cancer survivors, a longer smoking history and living with a smoker increased the odds of continued smoking after diagnosis, as did lower levels of social well‐being. The type of cancer diagnosed also impacted smoking cessation rates, with lung cancer survivors most likely to quit after diagnosis. These findings are similar to other populations and highlight that continued smoking after a cancer diagnosis is common, and research into the barriers associated with cessation should be explored in AA cancer survivors. Smoking cessation must remain a priority to health care providers as they care for the growing number of cancer survivors, particularly those from underrepresented groups or with lower socio‐economic resources, to improve the health of this population.

## 6 ETHICS STATEMENT

This study was approved by Wayne State University Institutional Review Board (IRB #050417M1F).

## CONFLICT OF INTEREST

No conflicts.

## AUTHOR CONTRIBUTIONS

Carly Malburg: Writing‐original draft, and writing‐review and editing. Juliana Fucinari: Formal analysis, writing‐original draft, and writing‐review and editing. Julie J. Ruterbusch: Supervision, formal analysis and writing‐review and editing. David M. Ledgerwood: Writing‐review and editing. Jennifer L. Beebe‐Dimmer: Writing‐review and editing. Ann G. Schwartz: Conceptualization, funding acquisition, methodology, supervision, visualization, and writing‐review and editing. Michele L. Cote: Conceptualization, supervision, and writing‐review and editing.
